# Mechanism of miR-7 mediating TLR4/TRAF6/NF-κB inflammatory pathway in colorectal cancer

**DOI:** 10.1007/s10142-024-01307-0

**Published:** 2024-02-05

**Authors:** Jianfeng Ren, Bing Han, Ping Feng, Gang Shao, Yunli Chang

**Affiliations:** 1 Department of Gastroenterology, Shanghai Fourth People’s Hospital Affiliated to Tongji University, Shanghai, China; 2 Department of Gastroenterology, Pudong New Area People’s Hospital, 490 Chuanhuan Nan Lu, Pudong New Area, Shanghai, China

**Keywords:** miR-7, TLR4, TRAF6, NF-κB, Colorectal cancer

## Abstract

This study is aimed at investigating the roles of Toll-like receptor 4 (TLR4) and microRNA-7 (miR-7) in colorectal cancer (CRC) development and progression. We assessed TLR4 and miR-7 expression in CRC cells and tissues using reverse transcription-quantitative polymerase chain reaction. The relationship between miR-7 and TLR4 was analyzed through dual luciferase reporter assays. MTT, wound healing, and cell invasion assays were conducted to examine the effects of TLR4 and miR-7 on CRC cell proliferation, migration, and invasion. Western blotting was used to explore the involvement of the TRAF6/NF-κB signaling pathway. miR-7 was underexpressed in CRC, while TLR4 levels were increased. miR-7 negatively regulated TLR4 expression and its knockdown enhanced CRC cell proliferation, migration, and invasion. TLR4 knockdown had the opposite effects. The TRAF6/NF-κB pathway was linked to TLR4’s role in tumor progression. miR-7 might inhibit TRAF6/NF-κB target a signaling pathway of TLR4 and promote CRC occurrence. miR-7 may therefore be used as a sensitive biomarker in CRC patients.

## Introduction

Worldwide, colorectal cancer is the third most common cancer, accounting for about 10% of all cancer cases, and is the second leading cause of cancer-related deaths. About 4.1% of men and women are estimated to be diagnosed with colorectal cancer at some point during their lifetime. The 5-year relative survival rate for colorectal cancer is about 65%. Annual increases in CRC incidence and mortality can be seen, especially in large and medium-sized cities, which have become an important public health problem in China (Watson and Collins 2011). However, a major challenge in the treatment of colorectal cancer is the development of therapeutic resistance. Cancer cells can become resistant to the drugs used in chemotherapy, making treatment less effective. And is as the result of the carcinogenesis of intestinal epithelial cells under the action of exogenous or endogenous factors. Among them, exogenous carcinogenic factors include high-fat diet, red meat diet, excessive drinking, obesity, and lack of exercise (Mao et al. 2021). The endogenous factors are mainly genetic predisposition. There are a variety of genes involved in the occurrence and development of CRC (Wang et al. 2022a). Some are oncogenes, which are highly expressed in CRC. For example, Kirsten Rat Sarcoma Viral Oncogene Homolog (KRAS), Phosphoinositide 3-kinase (PI3K), and Raf regulate cell differentiation by activating growth factors, and their mutations cause abnormal cell proliferation and activation. Some are tumor suppressor genes, such as Phosphatase and Tensin Homolog (PTEN) and Deleted in Colorectal Cancer (DCC), which are very low expressed in CRC, and their mutation and inactivation can stimulate tumor growth (Shen et al. 2021). The search for new therapeutic targets in colorectal cancer is ongoing. Microribonucleic acids (miRNAs) are endogenous functional single-stranded non-coding small molecule RNAs with a size of about 20–22 nt (Ferreira et al. 2022). By pairing with the mRNA bases of target genes, miRNA guides the silencing complex, degrades mRNA, hinders its translation, and engages in a variety of daily activities, like cell proliferating, organ, differentiation, tissue development, and tumor formation (Wang et al. 2022b). The discovery by Michael et al. (2003) that miR-145 as well as miR-143 expression levels in CRC were less than those in normal tissues, which detailed the function of miRNAs in CRC expression for the first time. As miRNAs inhibit tumor growth, miR-145 as well as miR-143 downregulates RAS and affects the function of this pathway (Yin et al. 2013). PI3K pathway activity is decreased by miR-194 through preventing Protein Kinase B (AKT) expression, further hindering cell proliferation (Zhao et al. 2014). miR-96 is upregulated in CRC, and it plays a role in downregulating p53 by targeting Tumor Protein P53 Inducible Nuclear Protein 1 (TP53INP1) (Gao and Wang 2015). miR-34a increases the activity of p53 by inhibiting Sirtuin 1 (SIRT1) (Yan et al. 2021). miR-320a directly targets SRY-Box Transcription Factor 4 (SOX4) and Forkhead Box M1 (FOXM1) and exerts a cancer-suppressive effect on CRC (Vishnubalaji et al. 2016). miR-214 expression was upregulated in colitis-associated CRC, while no significant difference was found between sporadic CRC and normal control samples. miR-214 indirectly activates NF-κB, a powerful inflammatory factor, thereby inhibiting the expression of PTEN and PDZ and promoting the formation of colitis-related CRC (Polytarchou et al. 2015). miR-7 is involved in Drosophila development (Chen et al. 2016). miR-7 has been found to have decreased expression in several malignant malignancies, CRC is included and regulates many oncogenic signal transduction pathways, pointing to the possibility that it functions as a tumor suppressor (Suto et al. 2015; Li et al. 2014). Thus, regulatory mechanism of miRNAs in CRC is very complex, which is worthy of further study and exploration.

Toll-like receptor 4 is a transmembrane protein whose cytoplasmic Toll/IL-1 receptor domain binds to ligands and initiates signaling cascades of inflammatory responses (Won et al. 2021; Drexler and Foxwell 2010). The concentration of TLRs increases under infection and inflammation (Oever et al. 2014). A recent study found that serum TLR4 was elevated in Non-Small Cell Lung Cancer (NSCLC) (Wei et al. 2016).

In this study, investigating the function of miR-7 in CRC and its underlying mechanisms was the goal. The findings indicated that miR-7 expression was considerably lower in CRC. By directly suppressing TLR4, over-expression of miR-7 inhibits CRC cell proliferation, migration, and invasion while also blocking the TRAF6/NF-κB pathway.

## Materials and methods

### Patients

Between January 2021 and January 2022, colorectal cancer patients at Shanghai Fourth People’s Hospital connected with Tongji University provided 30 samples of colorectal cancer tissues and matched neighboring non-cancer tissues. The included patients were aged 34 to 74, and mean age was 63 ± 8 years. By colonoscopy, all patients had colorectal cancer that was histologically verified. None had received chemotherapy or radiation. Patients were pathologically and clinically diagnosed with colorectal cancer. CRC specimens were obtained from patients after pathological diagnosis. Five-milliliter peripheral blood samples were amassed during hospitalization to detect CTCs. Moreover, patients, who underwent neoadjuvant chemotherapy/radiotherapy, were excluded. All tissue collections and experiments were reviewed and approved by the Institutional Review Boards of Tongji University. The studies were conducted in accordance with recognized ethical guidelines (Declaration of Helsinki). Informed consent was obtained from all of the participants.

### Cell culture

HCT116 (TCIIu), LoVo (TCIIu), HT29 (TCHu), SW620 (TCHf), and SW480 (TCHu) cell lines were purchased. Chinese Academy of Sciences sold the cell line NCM460. NCM460 and CRC cell lines were cultured in 37 °C incubator with 5% CO_2_ using Roswell Park Memorial Institute (RPMI) 1640 medium (Gibco, USA) containing 10% fetal bovine serum (FBS).

### Reagent

We obtained the TLR4 small interfering RNA, miR-7 mimic or inhibitor, and negative control from Guangzhou Rebo Biological Co., Ltd. (Guangzhou, China). GenScript created the p miR-Report TLR4 3′UTR WT and p miR-Report TLR4 3′UTR mutant sequences (Piscataway, NJ, USA).

### Cell transfection

Using Lipofectamine 2000 solution (12,566,014, Invitrogen), inhibitors of miR-7 (100 nM), negative control (100 nM), and miR-7 mimic (50 nM) were transiently transfected into SW620 cells. Following the requirements, the transfected cells were for 48 h with TLR4 siRNA (200 nmol/L) or miR-7 inhibitor plus TLR4 siRNA using oligofectamine transfection reagent. The sequences were as follows, miR-7 mimicF: 5′- UGGAAGACUAGUGAUUUUGUUGU -3′; R: 5′- AACAAAAUCACUAGUCUUCCAUU -3′, miR-7 inhibitorF: 5′- ACAACAAAAUCACUAGUCUUCCA -3′, miR-NC F: 5′- CAGUACUUUUGUGUAGUACAA -3′,si-NC F: 5′- UUCUCCGAACGUGUCACGUTT -3′; R: 5′- ACGUGACACGUUCGGAGAATT -3′, si-TLR4 F: 5′-UUGGAUUAUGAAAAUGCUCCA-3′; R: 5′-GAGGAUUUUCAUAAUGCAAAA-3.

### Dual luciferase reporter assays

For luciferase experiments, 24-well plates containing 2 × 10^4^ cells per well were seeded with 2 × 10^3^ cells and left incubating overnight. Transfected after 48 h, luciferase activity is measured using analysis of dual luciferase reporter kit (ABIN1000340, antibodies-online).

### Quantitative polymerase chain reaction for reverse transcription (RT-qPCR)

TRIzol (15,596,026, Invitrogen) reagent was used to extract total RNA. Amplification was performed using SYBR Green reagent and an ABI 7500 thermal cycler under the subsequent reaction circumstances: 95 °C for 40 cycles over 10 min, followed by 60 °C for 20 s and 72 °C for 35 s. 2^−ΔΔCt^ method was used, and results were normalized using the GAPDH expression. Utilizing the TaqMan microRNA kit (4,427,975, Invitrogen), miR-7 was measured. According to the instructions for using TaqMan MicroRNA Reverse Transcription Kit (4,366,596, Invitrogen), synthesize the cDNA of miR-7. Reaction conditions: 16 ℃, 30 min, 42 ℃, 30 min, 85 ℃, 5 min. In this study, U6B was used as the internal reference gene for miRNA detection. The primers for miR-7 were 5′- TGGAAGACTAGTGATTTTGTTG -3′;5′- ACGCTGGAAGACTAGTGATTTTG-3′, GAPDH5′- CGACAGCAGCCGCATCTT -3′; 5′- CCAATACGACCAAATCCGTTG -3′.

### MTT assay for cell proliferation

After transfected of miR-7 mimic, miR-7 inhibitor, negative control, and TLR4 siRNA, cell proliferating was assessed utilizing the MTT test ((3-(4-(5)-2-thiazolyl)-2-(5-diphenyl)-2-H tetrazolium), C0009S, Beyotime). SW620 cells, LoVo, and SW480 (3 × 10^3^ cells per well) were planted in 96-well culture plates for 24 h and then treated with 20 μL MTT (5 mg per mL) for 4 h at 37 degrees after 24, 48, and 72 h transfection. The growth liquid was then taken out, and each well received 150 μL of dimethyl sulfoxide, which was added for 30 min to dissolve all crystals. Using a spectrophotometer, the absorbance at 570 nm was measured (Ultrospec 2000).

### Wound healing assay

SW620 cells were inserted into 6-well plates. Then, 48 h after transfection, cell monolayers were scraped with the tip of a 10 μL sterile pipette. A microscope was used at 0 and 24 h after injury (IX711, Olympus Corporation, Tokyo, Japan) observed. The area for wound healing was quantitatively examined using Image J software.

### Assay for cell invasion

Transwell membrane filters were used in 24-well chambers where cell invasion tests were conducted (Corning, New York, USA). SW620 cells, LoVo, and SW480 were resuspended in 200 μL serum-free media forty-eight hours after transfection and then planted onto the plate’s upper chamber. In the lower chamber, 600 μL of culture media containing 10% FBS was put. Use cotton swab to remove the cells on the upper membrane following a 24-h incubation period. The invasion ability was assessed using the number the lower chamber of cells invading, and cells were counted at × 100 fold multiples in 5 random fields per well.

### Western blot analysis

The entire protein of the cells was extracted with protein lysis buffer, and a biquinolinic acid assay kit (P0012S, Beyotime, Haimen, China) was used to gauge the protein content. Using 10% SDS-PAGE (Sigma-Aldrich), protein samples (weighing 30 μg each) were analyzed. Afterward, transferred to membranes made of polyvinylidene fluoride. Block for 1 h at the temperature of the room in Tris-buffered saline/Tween-20 with 5% (w/v) skim milk, afterward, incubate the sample over one night at 4 °C centigrade with primary anti-CyclinD1 (#2922), anti-P21 (#37,543), anti-VEGF (#50,661), anti-E-Cadherin (#3195), anti-TLR4 (#38,519), anti-TRAF6 (#67,591), anti-NK-kB (#8943), and anti-p65 (#3033) with dilution 1:1000. We bought all our antibodies from Cell Signaling Technology. The membrane was then treated with horseradish peroxidase anti-rabbit IgG secondary antibody for 1 h at room temperature (1:2000, Cell Signaling Technology, Inc.). Using the Enhanced Chemiluminescence Detection System, protein band visualization. The National Institutes of Health’s ImageJ software, version 1.46, was used to calculate the bands’ individual grayscale values.

### Statistical analysis

Data were analyzed with SPSS software, version 13.0, and GraphPad Prism 9 software. The unpaired *t*-test was used for comparison between the two groups, repeated measures analysis of variance were employed, and then the Tukey post hoc analysis. *P* < 0.05 was used to determine statistical significance.

## Results

### Downregulated of miR-7 expression in cell lines and CRC tissues

**Fig. 1:**
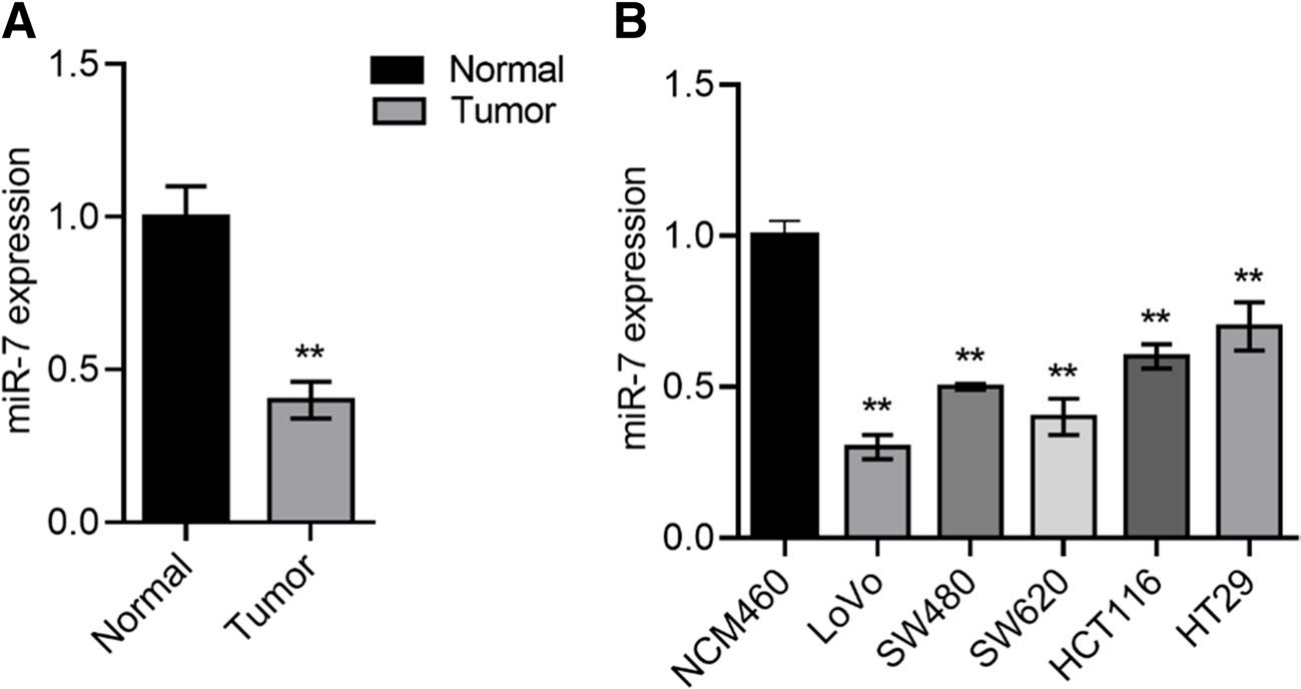
Downregulation of miR-7 expression in cell lines and CRC tissues. **A, B** miR-7 is found in colorectal cancer cell lines and tissues by qPCR. **P < 0.01

Results from RT-qPCR showed that miR-7 expression in tissues of CRC was much lower than that in normal tissues (*P* < 0.05, Fig. 1A). All five CRC cell lines drastically reduced the miR-7 expression (Fig. 1B). Indicating miR-7 may have a role in CRC development by suppressing tumor growth.

### Overexpression of miR-7 hindered the proliferation, migration, and invasion of CRC cells

**Fig. 2:**
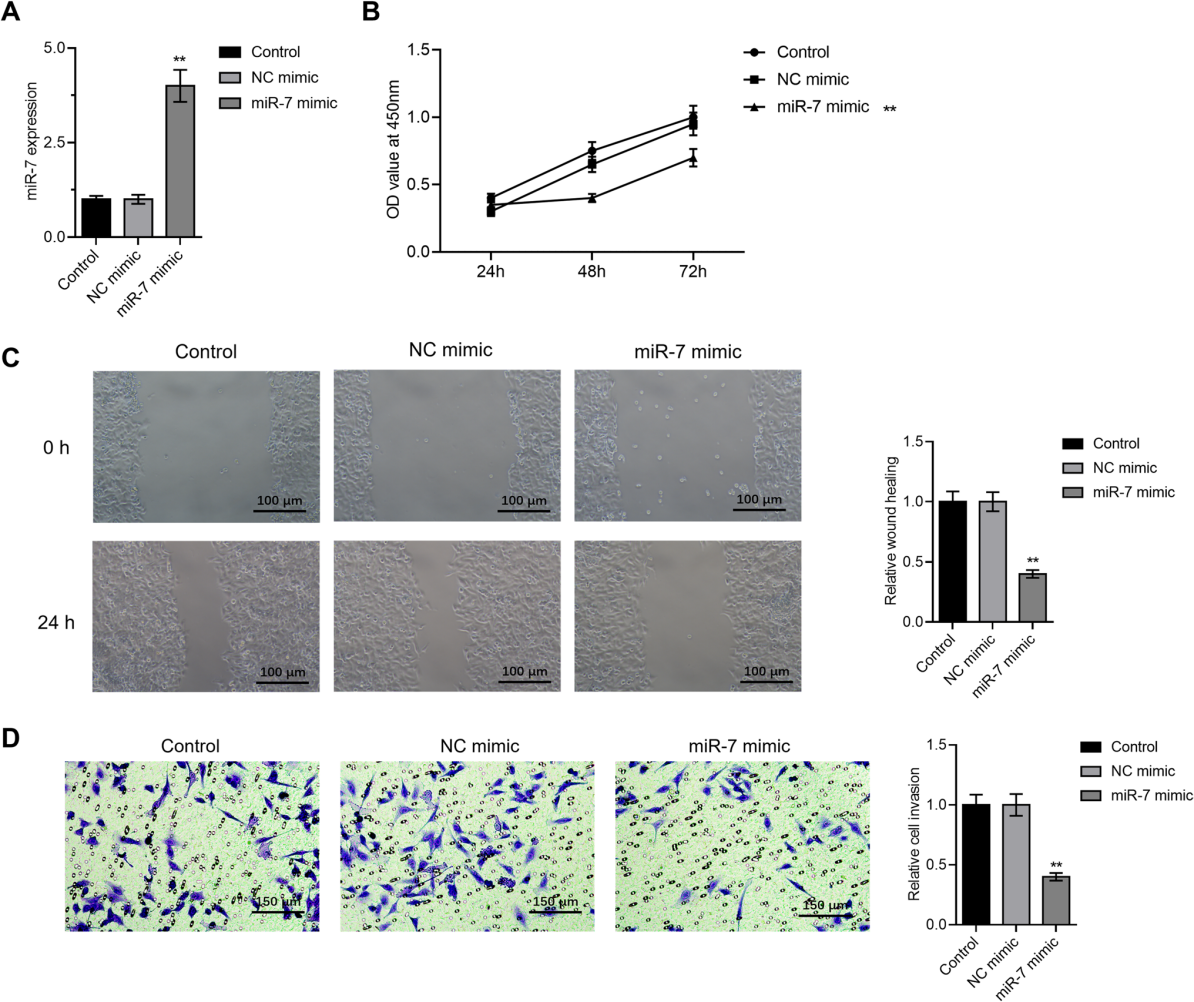
Overexpression of miR-7 hindered the proliferation, migration, and invasion of CRC cells. **A** Transfection efficiency of miR-7 was detected by qPCR. **B** MTT assay of cancer cell proliferation. **C** Test for cancer cell migration using scratches. **D** Cell invasion was discovered using the Transwell Assay (100X)

SW620 cell line was transfected with a miR-7 mimic or a negative management to examine the involvement of miR-7 in the formation of CRC. The transfection efficiency of miR-7 mimic was verified by RT-qPCR (Fig. 2A). As shown in Fig. 2C, the results of the wound healing experiment demonstrated that the miR-7 mimic hindered migration in comparison to the control. Additionally, Transwell experiments and MTT showed miR-7 overexpression prevented cellular proliferation and invasion (Fig. 2B, D). These findings imply that miR-7 contributes significantly to the progression of CRC.

### TLR4 is a direct miR-7 target gene

**Fig. 3:**
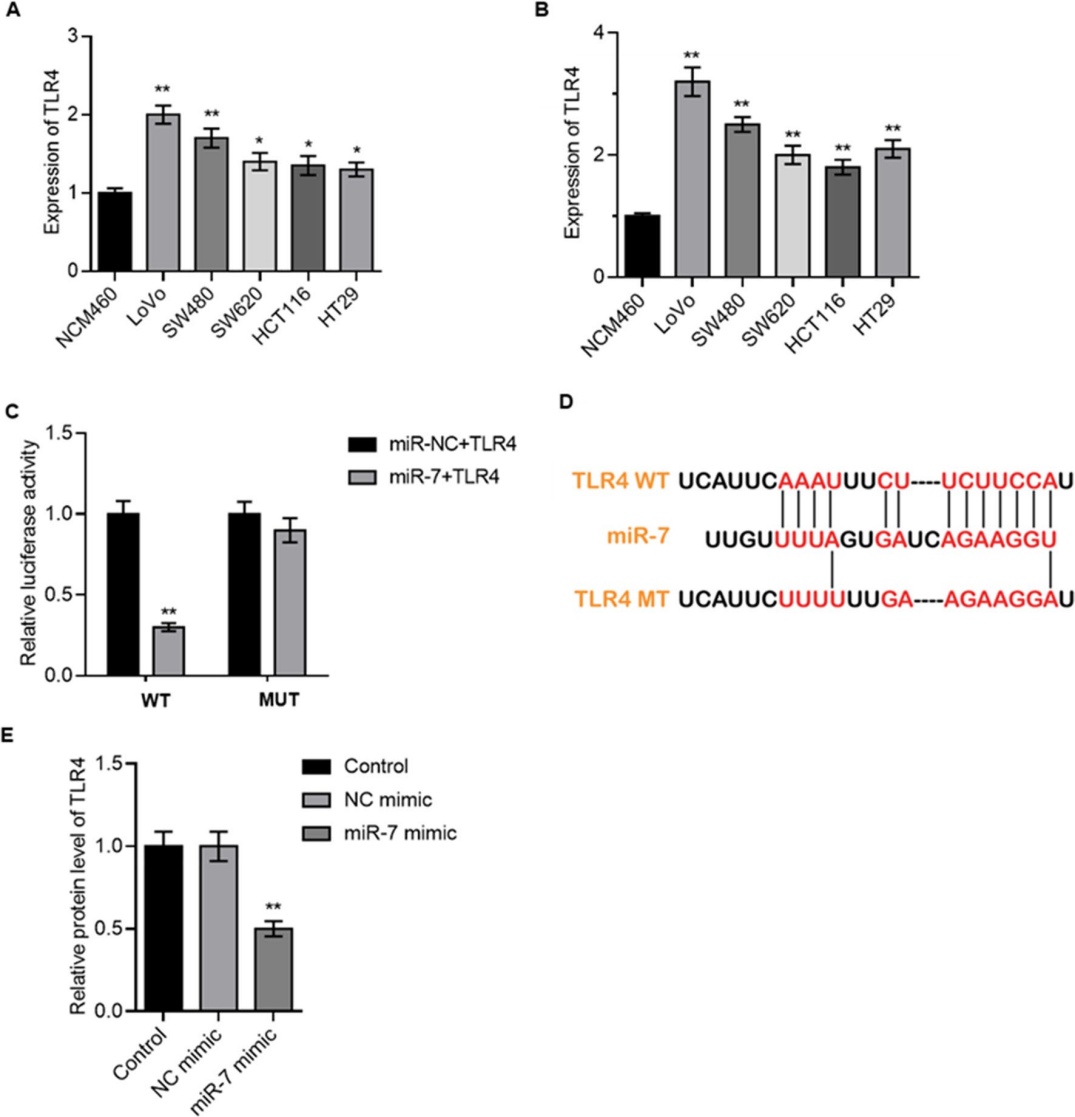
TLR4 is a direct miR-7 target gene. **A, B** The level of TLR4 in different colorectal cancer cell lines was detected. **C** Luciferase activity of cells in different groups. **D** Binding site between TLR4 and miR-7. **E** TLR4 protein level was detected

As previously described, in cell lines and CRC tissues, miR-7 expression was reduced (Fig. 1). TLR4 expression was later evaluated, and it was found to be increased in CRC cells (Fig. 3A, B). TLR4 was foreseen as a possible target of miR-7 by TargetScan, PicTar, and miRanda (Fig. 3C). According to Fig. 3D, in contrast to the luciferase activity of the MUT binding site was unaffected, the relative luciferase activity of TLR4 3’UTR WT was decreased by miR-7. TLR4 expression was also examined in relation to the effect of miR-7 mimics, and the findings revealed that miR-7 overexpression dramatically decreased TLR4 expression in CRC cells (*P* < 0.05, Fig. 3E). The transfection efficiency of the NC mimic cells was 76%, and miR-7 mimic cells were 81%.

### TLR4 regulates the proliferation, migration, and invasion of CRC cells

**Fig. 4:**
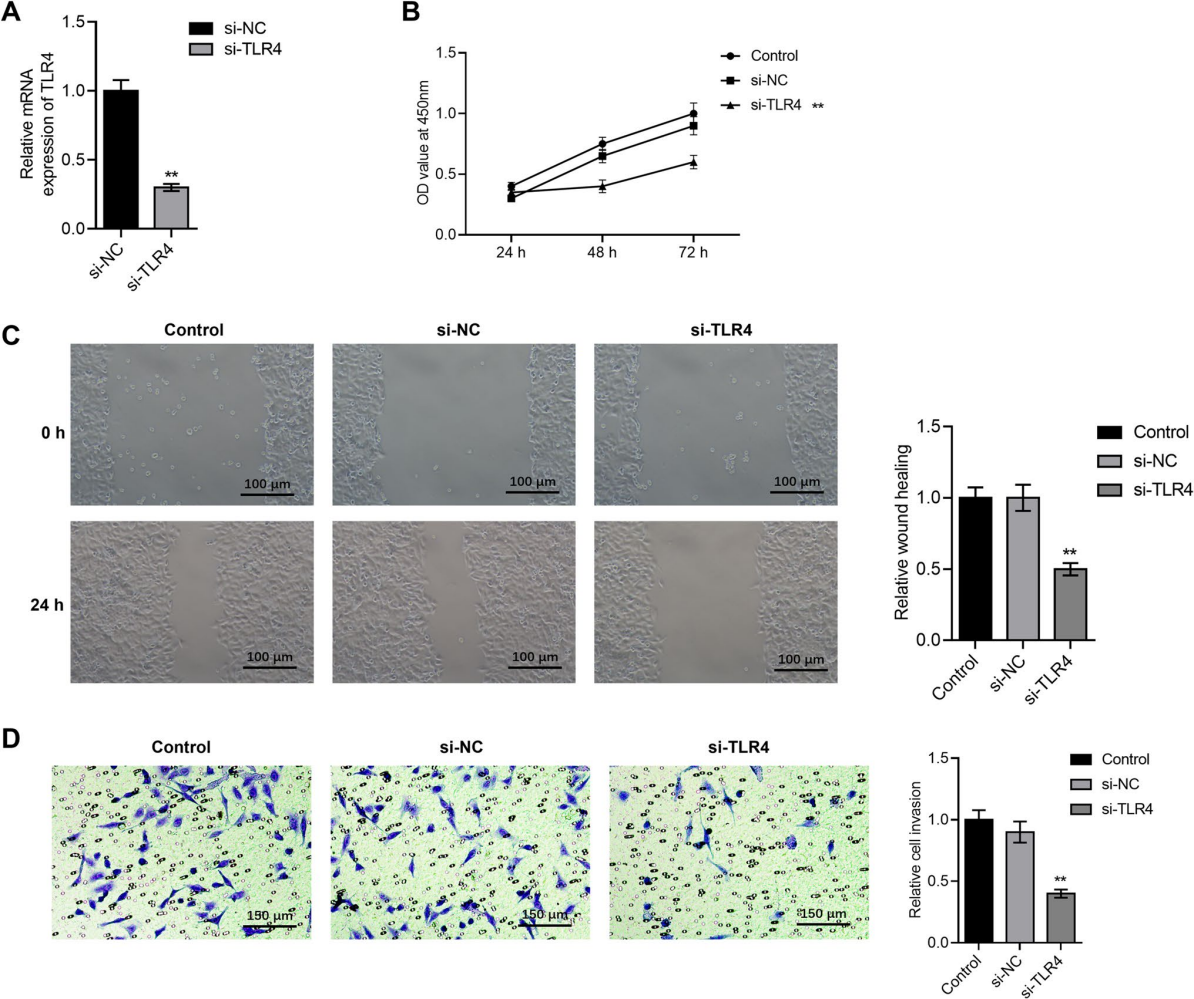
TLR4 regulates the proliferation, migration, and invasion of CRC cells. **A** TLR4 transfection efficiency was found using by qPCR. **B** To ascertain the effect of TLR4 expression on cancer cell proliferating, the MTT test was utilized. **C** Using scratch testing to determine TLR4 expression’s impact on cancer cell migration. **D** The impact of TLR4 on cell invasion was discovered using the Transwell test (100X)

To assess TLR4’s impact on CRC, we transfected TLR4 siRNA into SW620 cells. By using RT-qPCR, the interference effectiveness of si-RNA was confirmed (Fig. 4A). Then, using a wound-healing experiment, the impact of TLR4 knockdown on CRC cell migration was assessed. As shown in Fig. 4C, knockdown of TLR4 inhibited cell migration. MTT and Transwell assays further demonstrated that TLR4 knockdown inhibited cell proliferation and invasion (Fig. 4B, D). These results suggest a role for TLR4 in CRC progression.

### miR-7 regulates TRAF6/ NF-κB signaling pathway through TLR4

**Fig. 5:**
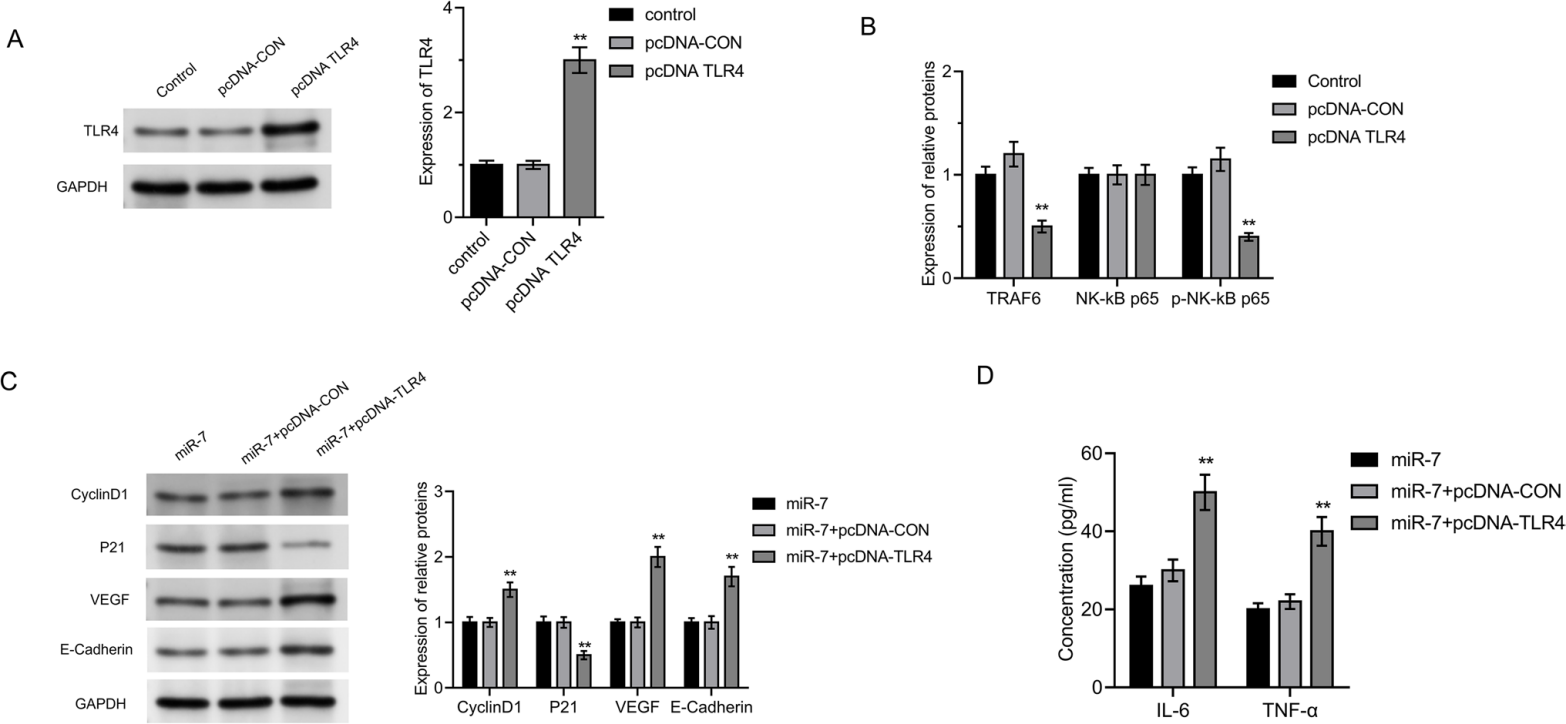
miR-7 regulates TRAF6/NF-κB signaling pathway through TLR4. **A** The efficiency of TLR4 plasmid was verified by Western blot. **B** The expression of TRAF6/NK-κB pathway protein was detected. **C** Proliferation and migration-related proteins were detected. **D** The concentration of inflammatory factors was measured using ELISA

TLR4 was increased in pcDNA-TLR4 group compared to pcDNA-CON group (Fig. 5A, P < 0.05). The levels of TRAF6, TLR4, and p-NK-κB p65 were lower than those in the miR-7 + pcDNA-con group (*P* < 0.05, Fig. 5B). In the miR-7 + pcDNA-TLR4 group, there were more moving cells, significantly higher CyclinD1 and VEGF protein levels, lower P21 and E-cadherin protein expression, and higher IL-6 and TNF-α mRNA levels (*P* < 0.05, Fig. 5C, D).

### TLR4 inhibits miR-7 function in CRC cells

**Fig. 6:**
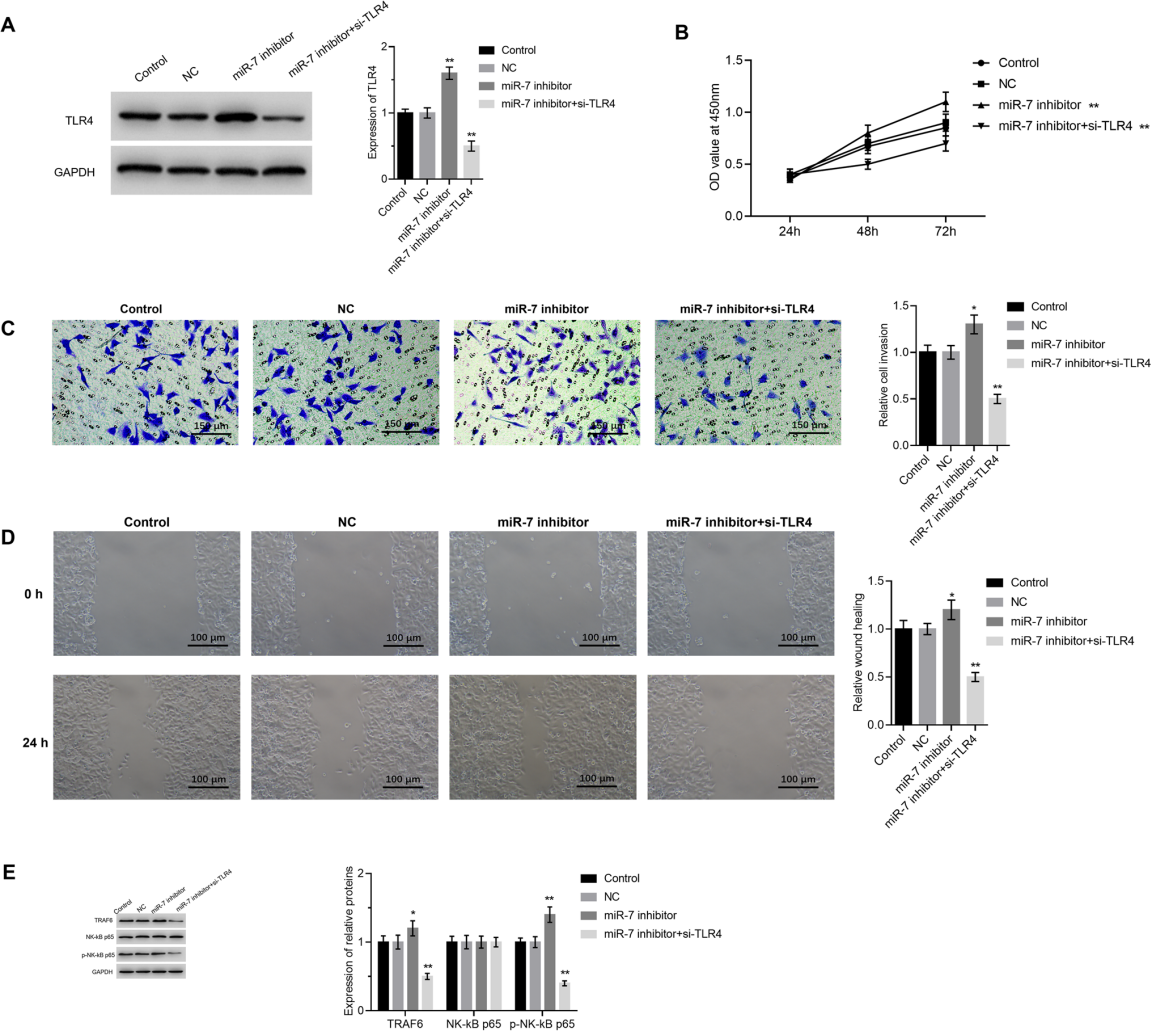
Inhibited miR-7 function by TLR4 in CRC cells. **A** TLR4 protein level was detected. **B** MTT assay of TLR4 cancer cell proliferation by miR-7. **C** The effect of TLR4 controlled by miR-7 on cancer cell invasion was discovered using the Transwell test (100X). **D** Scratch testing was used to identify TLR4’s role in miR-7-mediated regulation of cancer cell migration. **E** Using western blot to identify the expression of important components in the TRAF6/NK-κB signaling pathway

As shown in Fig. 6A, cells with inhibitor of miR-7 showed higher expression of TLR4. Then, co-transfection effect of TLR4 siRNA and miR-7 inhibitor was detected. In contrast to the miR-7 inhibitor-treated group, cell proliferation was reduced in co-transfected of cells with TLR4-siRNA and miR-7 inhibitor (*P* < 0.05, Fig. 6B). Additionally, miR-7 inhibitor group against miR-7 inhibitor and TLR4 siRNA group reduced migrating and invading (Fig. 6C, D), indicating that miR-7 may have an inhibitory effect on TLR4 that prevents CRC cells from proliferation, migration, and invasion. In addition, to clarify the potential mechanism of TLR4 in carcinogenesis, the activity of TRAF6/NF-κB pathway signaling molecules was detected. TRAF6, NK-κB P65, and p-NK-κB P65 levels in the TLR4-siRNA and miR-7 inhibitor groups were less than those in the group of miR-7 inhibitor (*P* < 0.05, Fig. 6E). According to these findings, miR-7 specifically targets TLR4 in CRC cells and prevents cell activation via TRAF6/NF-κB signaling.

## Discussion

CRC has a high incidence and causes social and economic burden. There is evidence that miRNAs contribute to the development of CRC. In this investigation, we discovered that CRC tissues and cell lines have downregulated miR-7 expression. By targeting TLR4 and blocking the TRAF6/NF-κB signaling pathway, miR-7 prevents CRC cells from proliferation, migration, and invasion their surrounding tissue.

Studies have indicated a correlation between increased levels of miR-7 and factors like tumor depth, invasion, and metastasis in various cancers, including CRC (Nagano et al. 2016). miR-7 is recognized as a tumor suppressor in CRC and other cancers (Li et al. 2014; Karadima et al. 2016). However, in CRC patients, its negative correlation with overall survival implies an oncogenic role (Suzuki et al. 2018). In non-small cell lung cancer cell lines, miR-7 induces apoptosis and restricts cell growth and migration. It also exhibits a tumor-suppressing function in CRC, proven to induce apoptosis by regulating cell cycle checkpoints in nude mice (Roychowdhury et al. 2021). Our study contributes to this understanding by showing that miR-7 expression is reduced in CRC cells. Importantly, we found that overexpressing miR-7 inhibits the proliferation and invasion of CRC cells, underscoring its potential as a therapeutic target. Bioinformatics studies predicted TLR4 as a potential target of miR-7, suggesting a functional interaction between them in CRC. Our preliminary examination revealed an inverse relationship between miR-7 and TLR4 expression in CRC cells, with TLR4 expression increasing as miR-7 levels decreased. Studies confirmed that TLR4 is involved in the process of mucosal cell death in intestinal I/R injury (Marques et al. 2014). Our study highlights the critical role of TLR4 in CRC progression and its interaction with miR-7, providing insights into potential therapeutic targets.

The expression level of TLR4 increases rapidly after intestinal I/R injury, along with TLR4 activation which can cause epithelial cell damage, I/R-mediated apoptosis, and tissue damage (Chen et al. 2020). TLR4 can further regulate the production of interferon and related cytokines through the MyD88 pathway (Varshney and Saini 2018). TRAF6, an adaptor protein in the TRAFs family, interacts with the TNF receptor, activating the NF-κB signaling pathway (Walsh et al. 2015). NF-κB and MAPKs are activated by TRAF6, thereby regulating the expression of downstream genes. Aberrant NF-κB activation via cytokine storm and tumor metastatic TME reconstitution has been observed in various human cancers (Luo et al. 2022; Lim et al. 2018). The exact dependency of miR-7 function on the TLR4 TRAF6/NF-κB signaling pathway was previously uncertain. Our study’s findings clarify this by showing that inhibiting miR-7 in CRC cell lines led to an increase in TRAF6/NF-κB signaling activity. Notably, this increase was countered by the knockdown of TLR4, indicating a critical interaction between miR-7, TLR4, and the TRAF6/NF-κB pathway in CRC.

In summary, our research demonstrates that miR-7 directly suppresses TLR4, potentially through the TRAF6/NF-κB signaling pathway, thereby regulating the proliferation, migration, and invasion of CRC cell lines. These findings indicate that TLR4 and miR-7 might be diagnostic and therapeutic targets for CRC.

## Data Availability

Data are stored in the zenodo database, 10.6084/m9.figshare.c.7075478.v1.
